# Impact of Inflammatory Bowel Disease (IBD) and IBD Medications on Risk of Hyperlipidemia and *in vitro* Hepatic Lipogenic-Related Gene Expression: A Population-Based Cohort Study

**DOI:** 10.3389/fmed.2022.910623

**Published:** 2022-06-13

**Authors:** Ni Tien, Tien-Yuan Wu, Cheng-Li Lin, Chia-Jui Wu, Chung-Y Hsu, Yi-Jen Fang, Yun-Ping Lim

**Affiliations:** ^1^Department of Laboratory Medicine, China Medical University Hospital, Taichung, Taiwan; ^2^Department of Medical Laboratory Science and Biotechnology, China Medical University, Taichung, Taiwan; ^3^Department of Pharmacy, Taichung Tzu Chi Hospital, Buddhist Tzu Chi Medical Foundation, Taichung, Taiwan; ^4^Department of Pharmacology, School of Medicine, Tzu Chi University, Hualien, Taiwan; ^5^Management Office for Health Data, China Medical University Hospital, Taichung, Taiwan; ^6^Department of Pharmacy, College of Pharmacy, China Medical University, Taichung, Taiwan; ^7^Graduate Institute of Biomedical Sciences, China Medical University, Taichung, Taiwan; ^8^Research Center for Environmental Medicine, Kaohsiung Medical University, Kaohsiung, Taiwan; ^9^Ph.D. Program in Environmental and Occupational Medicine, College of Medicine, Kaohsiung Medical University and National Health Research Institutes, Kaohsiung, Taiwan; ^10^Graduate Institute of Clinical Medicine, Department of Environmental Health, Kaohsiung Medical University, Kaohsiung, Taiwan; ^11^National Institute of Environmental Health Sciences, National Health Research Institutes, Zhunan, Taiwan; ^12^Digestive Disease Center, Show Chwan Memorial Hospital, Changhua, Taiwan; ^13^Department of Internal Medicine, China Medical University Hospital, Taichung, Taiwan; ^14^Department of Medical Research, China Medical University Hospital, Taichung, Taiwan

**Keywords:** inflammatory bowel disease (IBD), IBD medications, hyperlipidemia, Longitudinal Health Insurance Database (LHID), lipogenesis

## Abstract

Patients with inflammatory bowel disease (IBD) present a higher risk of developing cardiovascular diseases (CVDs) due to chronic inflammation, which plays an essential role in atherogenesis. Hyperlipidemia is another risk factor for CVDs; however, the association between IBD, IBD medications, and hyperlipidemia remains controversial. We conducted a nationwide, population-based, retrospective, cohort study to examine the effect of IBD and IBD medications on the risk of developing hyperlipidemia. The effects of IBD medications on the expression of lipogenesis-related hepatic genes were also evaluated. We obtained data from the Longitudinal Health Insurance Database of Taiwan from patients with new-onset IBD and a comparison cohort of patients without IBD. A Cox proportional hazards regression model was used to analyze the difference in the risk of developing hyperlipidemia between the two cohorts. We also examined the influence of IBD medications on the expression of lipogenesis-related hepatic genes. After adjusting for comorbidities and confounding factors, the case group (*N* = 14,524) had a higher risk for hyperlipidemia than the control group (*N* = 14,524) [adjusted hazards ratio (aHR), 2.18]. Patients with IBD that did not receive IBD medications exhibited a significantly higher risk of hyperlipidemia (aHR, 2.20). In those treated with IBD medications, the risk of developing hyperlipidemia was significantly lowered than those without such medications (all aHR ≤ 0.45). Gene expression analysis indicated that IBD medications downregulated the expression of lipogenesis-related genes. Screening blood lipids in IBD patients is needed to explore the specific role and impact of IBD medications in the development of CVD.

## Introduction

Inflammatory bowel diseases (IBDs) have no cure and are characterized by chronic, recurrent exacerbation and intestinal inflammation, resulting in altered gut functions (1). IBDs consist of Crohn's disease (CD) and ulcerative colitis (UC). The fundamental causes of these autoimmune diseases include the interplay between genetic and environmental factors, excluding pathogenic infections (2). IBD diagnoses depend on multiple factors, including clinical, endoscopic, radiological, and histological features, but not infectious etiology (1). In Taiwan, crude CD and UC incidences increased from 0.17 to 0.47 and 0.54 to 0.95 new cases per 100,000 persons, respectively, between 2001 and 2015. Moreover, CD and UC prevalence increased from 0.6 to 3.9 and 2.1 to 12.8 cases per 100,000 persons, respectively, within the same time frame. The male-to-female ratio in the study samples were 2.19 for CD and 1.62 for UC (3–5). Meanwhile, IBD prevalence in the USA is estimated to be 1.1–3 million adults (6). In the Western world, IBD prevalence is approximately 50–200 and 120–200 cases per 100,000 persons for CD and UC, respectively (7). J. Cosnes, C. Gower–Rousseau, P. Seksik, and A. Cortot, “Epidemiology and natural history of inflammatory bowel diseases,” *Gastroenterology*, vol. 140, no. 6, pp. 1785–1794.e4, 2011. View at: *Publisher Site*. UC, as a chronic and recurrent intestinal disease, is mainly an autoimmune disease caused by genetic–environmental interactions, rather than colonic colitis caused by general bacterial and viral infections.IBD cases increase annually, and drugs and changes to the environment can improve patient quality of life. Medications remain the main therapeutic strategy for IBD and to relieve inflammation. In addition, surgery can be introduced if medications are unsuccessful or result in serious adverse reactions (1).

IBDs coincide with clotting abnormalities and vascular-related comorbidities, such as deep vein thrombosis, portal vein thrombosis, and ischemic vascular diseases (8). It has been reported that patients with IBD have a venous thromboembolism (VTE) risk 1.7–5.9 times greater than the general population. This has been found to affect 0.55–6.15% of patients with IBD, and the overall prevalence of VTE in IBD subjects was estimated as 1–8% (9). Moreover, VTE-associated mortality is twice as high in patients with IBD than in the general population (10). Further, a meta-analysis demonstrated that IBD is associated with an 18% higher risk of CVD (11), and the risk is higher for females than males [adjusted odds ratio (aOR), 1.28] (8). Additionally, the risk of mesenteric ischemia is increased 3.4-fold, and that of VTE is increased 1.4-fold (12).

The relationship between IBD and hyperlipidemia risk should be evaluated. Moreover, further research is warranted to devise therapeutic modalities to prevent hyperlipidemia and consequently decrease the CVD risk for IBD patients. Preventive or therapeutic strategies can also be developed to identify the pathogenic causes of these complications (9, 11, 13–19). Hyperlipidemia, a well-established CVD risk factor (20), is defined as abnormal lipid levels with total cholesterol (TC) ≥ 200 mg/dL, triglycerides (TGs) ≥ 150 mg/dL, high-density lipoprotein cholesterol (HDL-C) ≤ 40 mg/dL, and low density lipoprotein cholesterol (LDL-C) ≥ 130 mg/dL. Hyperlipidemia can originate from genetics, diet, lifestyle, metabolic disorders, and other diseases (21). Additionally, the degree of hyperlipidemia correlates with CVD severity and can predict prognosis. For example, patients with IBD often have changing blood lipid profiles, similar to those reported in CVD, frequently caused by IBD medications. Thus, risk factor modulation is needed to reduce blood lipid levels and CVD risk (8).

In contrast to lipid levels in hyperlipidemia, patients with IBD have low levels of TC and HDL-C and high levels of LDL-C and TGs (22). The exact mechanism underlying these altered levels is unknown; however, active inflammation and changes to lipid, apolipoprotein, and lipoprotein profiles via altered lipid *de novo* synthesis and degradation might play a role (22–24). Despite these lipid levels, patients with IBD have an increased risk of CVD (25), resulting in a “lipid paradox”. However, studies showing these results are limited by small sample sizes. Moreover, the relationship between IBD therapeutics and reduced CVD risk remains controversial (26). Thus, we conducted a long-term, retrospective study with a large cohort to evaluate patients with IBD and the effects of their medications.

The mechanisms underlying the increased risk of CVD in patients with IBD are under investigation; however, systemic inflammation might play a role. Different drugs are selectively and broadly used to inhibit inflammation in IBD, controlling the active disease and inhibiting remission. Pharmacological treatment for IBD can be divided into four main classes, (1) aminosalicylates (5-aminosalicylic acid derivatives), (2) corticosteroids, (3) immunosuppressants, and (4) monoclonal antibodies (26). 5-Aminosalicylates are the most widely used and are ideal for IBD with mild to moderate symptoms mainly by blocking prostaglandin and leukotriene production (27). Corticosteroids are also used for reducing inflammation, however, they frequently have side effects dependent on the dose and treatment duration (28). Immunosuppressive and immunoregulatory agents could include the suppression of a specific subgroup of T cells, achieving a therapeutic response after a prolonged period (28). Therefore, these drugs are only useful for long-term control, rather than acute disease. Biologics are groups of monoclonal antibodies for patients with a reduced response to IBD drugs with small molecules. Several anti-tumor necrosis factor (TNF) therapies could inhibit TNF production by macrophages through altered regulatory peptide expression with IBD, which might lead to monocyte apoptosis (28). In addition, biologics can block leukocyte migration by blocking integrin adhesion molecules. Nevertheless, when prolonged treatment results in complications or adverse events, surgery can also be performed.

The liver is responsible for most processes involved in lipid homeostasis, including lipogenesis and blood lipid balancing. Therefore, any changes in hepatic lipid metabolism might affect the balance and homeostasis of blood lipid levels and could result in the development of non-alcoholic fatty liver disease (NAFLD) (29, 30). Liver X receptor alpha (LXRα), a nuclear receptor activated by ligands and act as transcription factor, is highly and specifically expressed in liver, responsible for lipid metabolism and *de novo* synthesis and excretion of cholesterol (31). LXRα activation results in the development of steatosis, which is mediated by the hepatic lipogenic pathway, primarily through sterol regulatory element binding protein 1 (SREBP-1c) (32). In addition, hepatic expression of LXRα, SREBP-1c, and their target genes, was found to be significantly upregulated in liver biopsies from NAFLD patients (33). Ultrasonographic monitoring revealed hepatic steatosis in approximately 50% of patients with associated with hypertriglyceridemia (29). Hyperlipidemia is commonly associated with NAFLD and is an independent risk factor of atherogenic dyslipidemia based on various clinical studies (33). Thus, the most important role of LXRα is the maintenance of lipid homeostasis as it regulates the balance of lipid-metabolism genes.

Further studies are warranted to determine how treatments for IBD affect hyperlipidemia risk. Thus, we performed a nationwide, population-based, cohort study to evaluate the risk of hyperlipidemia in patients with IBD from the 2000 to 2012 National Health Insurance Research Database (NHIRD) compared to that in the general population in Taiwan. We also examined the impact of IBD medications on the risk of hyperlipidemia and the expression of lipogenesis-related genes in differentiated HepaRG cells. We show that patients with IBD are more likely to have hyperlipidemia than those without IBD.

## Materials and Methods

### Data Source

We obtained data from the NHIRD. The database covers >99% of the population of 23 million in Taiwan and was constructed using comprehensive inpatient and outpatient health care information, including demographic data, diagnostic codes, and prescription details. The dataset from the NHIRD is a subset of the Longitudinal Health Insurance Database, which comprises data of one million randomly sampled beneficiaries enrolled in the NHI program. The International Classification of Diseases, Clinical Modification (ICD-9-CM, procedure code 555 and 556) was used as the disease diagnostic tool. This study was approved by the Central Regional Research Ethics Committee of China Medical University, Taichung, Taiwan (CMUH-104-REC2-115-R5).

### Study Population

In this population-based cohort study, we established an IBD cohort and a non-IBD cohort of patients enrolled in the database from January 1, 2000 to December 31, 2012 to compare their risk of hyperlipidemia. The index date of the case group (IBD cohort) was defined as the date of the first diagnosis of IBD and that of the control group (as the non-IBD cohort) was a random date during the study period. We excluded patients who had a history of hyperlipidemia before the index date. According to age (5-year intervals), gender, and the index year, the cohorts were frequency matched at a 1:1 ratio. The end date of the follow-up period was the onset of hyperlipidemia, death, or the end of study period (December 31, 2013), whichever came first. The primary outcome of the study was an individual event of hyperlipidemia (ICD-9-CM code 272) is defined as increased serum fasting levels of TC (≥200 mg/dL), LDL-C (≥130 mg/dL), or TG (≥150 mg/dL), and elevations of fasting TC concentration, which may or may not be associated with the elevated TG concentration, and decreased of HDL-C. To avoid subjects being mistakenly diagnosed or mistakenly coded as hyperlipidemia cases, we therefore defined patients with at least two claims for outpatient care and/or one hospitalization visit to ensure the validity of diagnosis. We selected potential confounders based on the previous research for multivariable analysis, including age, sex, and comorbidities of type 2 diabetes mellitus (T2DM) (ICD-9-CM 250), obesity (ICD-9-CM 278), coronary artery disease (CAD) (ICD-9-CM 410–414), hypertension (ICD-9-CM 401–405), and chronic kidney disease (CKD) (ICD-9-CM 585, 586). Further analysis was performed to investigate the effect of IBD treatment available in Taiwan on the risk of hyperlipidemia compared to non-IBD controls, IBD without medical treatment, and IBD without surgical treatment.

### Chemicals and Cell Culture

All chemicals were purchased from Sigma-Aldrich (St. Louis, Missouri, USA) and were of the highest-purity grade available. Chemicals were dissolved in dimethyl sulfoxide (DMSO) at appropriate concentrations before use. Human hepatoma HepaRG^TM^ cells were purchased from Thermo Fisher Scientific (Waltham, Massachusetts, USA). Frozen cells were thawed and maintained in Williams' E medium (Sigma-Aldrich) supplemented with 10% Fetal Clone^TM^ II serum (Hyclone^TM^, GE Healthcare, Chicago, Illinois, USA), 1 × L-glutamine, 5 μg/mL human insulin, and 50 μM hydrocortisone hemisuccinate without antibiotics for 2 weeks. Next, the medium was replaced with the same medium plus 2% DMSO for two additional weeks to induce differentiated hepatocyte-like properties. Cells were cultured in a humidified atmosphere of 5% CO_2_ at 37°C. Cell viability was assessed using *p*-nitrophenylphosphate in an acid phosphatase assay (ACP), as previously reported (34).

### RNA Isolation and Quantitative Real-Time Polymerase Chain Reaction (qRT-PCR)

To evaluate the effects of IBD medications (corticosteroids, immunomodulators, and aminosalicylates) on hepatic lipogenesis-related gene expression, mRNA levels were measured. Total RNA was extracted from differentiated HepaRG cells under various treatment conditions using a Direct-zol^TM^ RNA MiniPrep kit (ZYMO Research, Irvine, CA, USA) according to the manufacturer's protocol. The quantity and purity of RNA were confirmed by calculating the ratio of the absorbance at 260 nm to the absorbance at 280 nm. Total RNA (1 μg) was subjected to synthesis of first-strand cDNA using a MultiScribe^TM^ reverse transcriptase kit (ThermoFisher Scientific). Expression of *SREBP-1c, SCD, FAS*, adenosine 5′-triphosphate citrate lyase (*ACLY), ACC, LXR*α, and β*-actin* was analyzed by qRT-PCR using Luminaris Color HiGreen qPCR master mix (ThermoFisher Scientific) in the StepOnePlus^TM^ Real-Time PCR System following the standard procedure. Each pair of specific primers used for RT-PCR analysis is listed in Table 1. The amount of target cDNA in each sample was calculated by determining a fractional PCR threshold cycle number (Ct value). The relative mRNA levels were normalized to those of β*-actin*, and the target cDNA expression was calculated as follows: 2^−(*Cttargetgene*−Ct*β-actin*)^. Data are presented as fold-change compared to the control group.

**Table 1:** Sequences of PCR primers.

**Gene**	**Species**	**Forward primer (5′-3′)**	**Reverse primer (5′-3′)**
*SREBP-1c*	human	CGC TCC TCC ATC AAT GAC AA	TGC AGA AAG CGA ATG TAG TCG AT
*SCD*	human	CCG ACG TGG CTT TTT CTT CT	GCG TAC TCC CCT TCT CTT TGA C
*FAS*	human	ACA TCA TCG CTG GTG GTC TG	GGA GCG AGA AGT CAA CAC GA
*ACLY*	human	GTG TGG ACG TGG GTG ATG TG	TTG ATG TCC TCA GGA TTC AGT TTC
*ACC*	human	CTC TTG ACC CTG GCT GTG TAC TAG	TGA GTG CCG TGC TCT GGA T
*LXRα*	human	CGA TC GAG GTG ATG CTT CTG	GGC AAA GTC TTC CCG GTT AT
*β-actin*	human	CCT GGC ACC CAG CAC AAT	GCC GAT CCA CAC GGA GTA CT

### Statistical Analysis

We evaluated the frequency and percentage of each categorical variable and the mean and standard deviation (SD) of each continuous variable. A chi-squared test was used to examine the differences of the demographic categorical variables between the IBD and non-IBD cohorts. Student's *t*-test was used to measure the association of continuous demographic variables between the two cohorts. To address the concern of constant proportionality, we examined the proportional hazard model assumption using a test of scaled Schoenfeld residuals. Results showed that there was no significant relationship between Schoenfeld residuals for IBD and follow-up time (*p*-value = 0.68) in the model evaluating the hyperlipidemia risk. Stratified Cox models were used to estimate the risk of hyperlipidemia by sex, age, and comorbidity between the two cohorts. The aHR was obtained for age, sex, and comorbidities of T2DM, obesity, CAD, hypertension, and CKD disease through multivariable analysis. The Kaplan–Meier method was applied to estimate cumulative incidence curves of hyperlipidemia in both cohorts, with significance based on the log-rank test. Analyses were performed in SAS software, version 9.4, and survival curves were drawn using R software.

For *in vitro* studies, data obtained from separate measurements were reported as the mean ± standard error (SE). The *P*-value for each experimental comparison was determined using analysis of variance, followed by the least significant difference test for multiple comparisons. All *P*-values were determined relative to the control group, as indicated in the figures. All statistical analyses were performed using SPSS for Windows, version 20.0 (IBM SPSS, Armonk, NY, USA). *P* < 0.05 was considered statistically significant.

## Results

### Baseline Characteristics: Demographic and Association Findings

**Figure 1:**
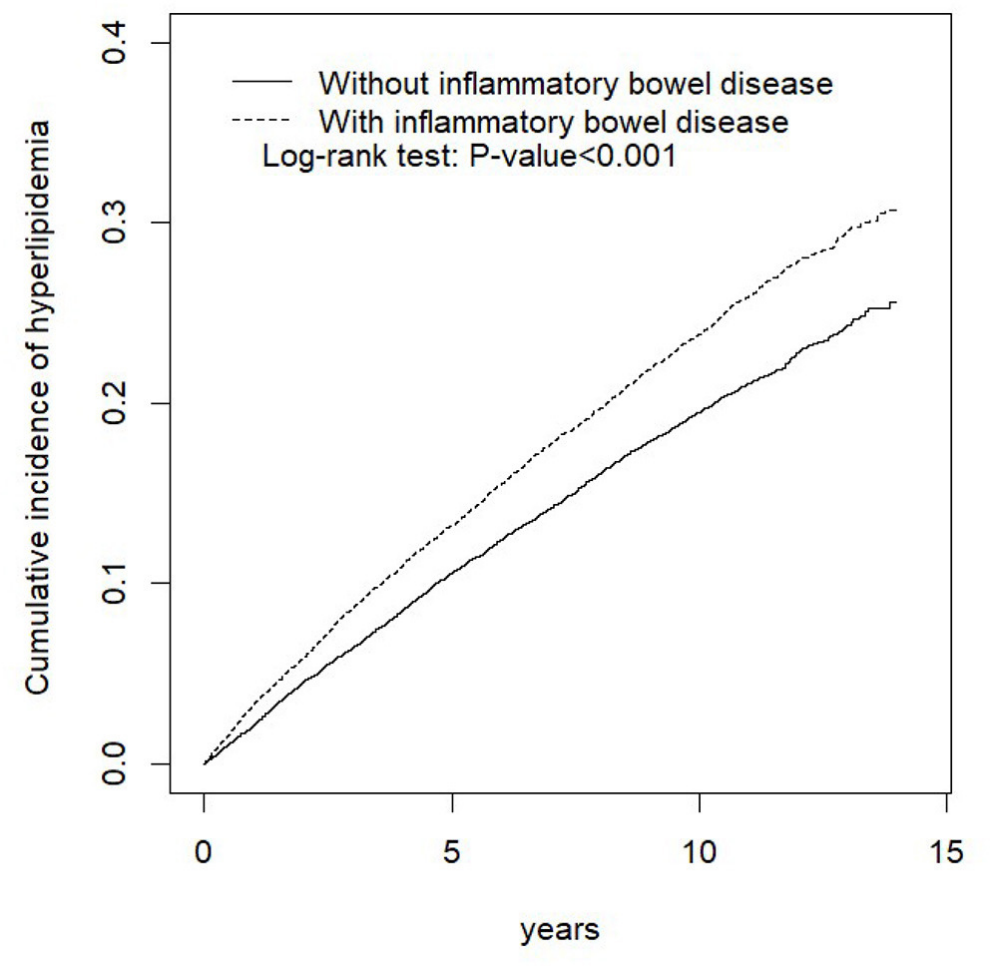
Cumulative incidence of hyperlipidemia compared between the cohort with and without inflammatory bowel disease (IBD) using the Kaplan–Meier method.

Table 2 shows the baseline characteristics of the study population from 2000 to 2013. After propensity score matching, and calculating the real-world database power as 0.9996, this study included 14,524 patients with and without IBD, respectively. Among the types of IBD, 1,362 (9.38%) and 13,162 (90.6%) patients had UC and CD, respectively. The mean ages for the IBD and non-IBD cohorts were 44.3 (SD = 16.4) and 43.9 (SD = 16.7) years, respectively. No significant differences were observed between the two cohorts in sex, age, or comorbidity of CKD. Patients with IBD were at higher risk of T2DM, obesity, CAD, and hypertension. Regarding treatment for patients with IBD, 68.6%, 0.97%, 3.60%, 0.03%, and 1.46% of patients received corticosteroids, immunomodulators, aminosalicylates, monoclonal antibodies, and surgical treatment, respectively. As shown in Figure 1, the Kaplan–Meier plots revealed that the cumulative incidence curve of hyperlipidemia showed a significantly higher risk in patients with IBD than those without.

**Table 2:** Demographic characteristics, comorbidities, and medications in patient with and without inflammatory bowel disease (IBD).

**Variable**	**IBD**		***p*-value**
	**No**	**Yes**	
	***N* = 14,524**	***N* = 14,524**	
	***N* (%)**	***N* (%)**	
**Type of IBD**			
Ulcerative colitis (UC)		1,362 (9.38)	
Crohn's disease (CD)		13,162 (90.6)	
Sex			0.99
Female	7,820 (53.8)	7,820 (53.8)	
Male	6,704 (46.2)	6,704 (46.2)	
Age, mean (SD)	43.9 (16.7)	44.3 (16.4)	0.08
Stratify age			0.99
≤49	9,765 (67.2)	9,765 (67.2)	
50–64	2,798 (19.3)	2,798 (19.3)	
65+	1,961 (13.5)	1,961 (13.5)	
**Comorbidity**			
Type 2 diabetes mellitus (T2DM)	394 (2.71)	453 (3.12)	0.04
Obesity	101 (0.70)	132 (0.91)	0.04
Coronary artery disease (CAD)	1,055 (7.26)	1,479 (10.2)	< 0.001
Hypertension	2,341 (16.1)	2,808 (19.3)	< 0.001
Chronic kidney disease (CKD)	120 (0.83)	143 (0.98)	0.15
**Medications**			
Corticosteroids		9,959 (68.6)	
Immunomodulators		141 (0.97)	
Aminosalicylates		523 (3.60)	
Monoclonal antibody and small molecule		5 (0.03)	
Surgical treatment		212 (1.46)	

^#^*Chi-Square Test*.

*Two sample T-test*.

Table 3 displays the stratified analyses of the risk of hyperlipidemia by sex, age, and comorbidity between the two cohorts. Compared with the control cohort, the IBD cohort faced a 2.18-fold higher risk of hyperlipidemia (95% CI, 2.03–2.34). Compared with that in the control cohort, UC resulted in a 2.60-fold increased hyperlipidemia risk (95% CI, 2.06–3.28), and the CD group was associated with a 2.15-fold increased hyperlipidemia risk (95% CI, 1.99–2.32). Moreover, females had a 2.10-fold higher risk of hyperlipidemia in the IBD cohort after controlling for other factors (95% CI, 1.90–2.32), while the risk for males in the IBD cohort was 2.25-fold (95% CI, 2.03–2.49). Among patients aged ≤ 49 years, the IBD cohort had a 2.00-fold higher risk of hyperlipidemia than the non-IBD cohort (95% CI, 1.81–2.21). Similarly, those aged 50–64 years had a 2.10-fold higher risk of hyperlipidemia in the IBD cohort (95% CI, 1.86–2.37). Additionally, among those aged ≥65 years, the IBD cohort had a 2.78-fold higher risk of hyperlipidemia (95% CI, 2.34–3.31). Moreover, among patients without comorbidities, the IBD cohort had a 2.06-fold higher risk of hyperlipidemia compared to the non-IBD cohort. Meanwhile, patients with comorbidities in the IBD cohort had a 2.25-fold higher risk of hyperlipidemia than those in the non-IBD cohort.

**Table 3:** Comparison of incidence (CI) and hazard ratio (HR) of hyperlipidemia stratified by sex, age, and comorbidities between with and without inflammatory bowel disease (IBD).

	**IBD**							
	**No**			**Yes**				
**Variable**	**Event**	**PY**	**Rate^#^**	**Event**	**PY**	**Rate^#^**	**Crude HR (95% CI)**	**Adjusted HR^*†*^ (95% CI)**
All	2,345	107,920	21.7	2,927	106,110	27.6	1.27 (1.20, 1.34)^***^	2.18 (2.03, 2.34)^***^
**Type of IBD**								
UC	252	10,484	24.0	310	9,904	31.3	1.30 (1.10, 1.54)^***^	2.60 (2.06, 3.28)^***^
CD	2,093	97,436	21.5	2,617	9,6213	27.2	1.27 (1.20 1.34)^***^	2.15 (1.99, 2.32)^***^
**Sex**								
Female	1,301	59,279	22.0	1,540	58,317	26.4	1.20 (1.12, 1.29)^***^	2.10 (1.90, 2.32)^***^
Male	1,044	48,641	21.5	1,387	47,793	29.0	1.35 (1.25, 1.47)^***^	2.25 (2.03, 2.49)^***^
**Stratify age**								
≤49	1,128	77,553	14.5	1,520	76,695	19.8	1.36 (1.26, 1.47)^***^	2.00 (1.81, 2.21)^***^
50–64	822	18,830	43.7	952	18,262	52.1	1.19 (1.09, 1.31)^***^	2.10 (1.86, 2.37)^***^
65+	395	11,537	34.2	455	11,153	40.8	1.19 (1.04, 1.36)^*^	2.78 (2.34, 3.31)^***^
**Comorbidity^‡^**								
No	1,429	91,362	15.6	1,742	85,918	20.3	1.30 (1.21, 1.39)^***^	2.06 (1.88, 2.26)^***^
Yes	916	16,558	55.3	1,185	20,191	58.7	1.06 (0.97, 1.16)	2.25 (2.01, 2.51)^***^

# *incidence rate, per 1,000 person-years; Crude HR, crude hazard ratio*.

† *Multivariable analysis including age, sex, and comorbidities of T2DM, obesity, CAD, hypertension, and CKD*.

‡ *Patients with any one of the comorbidities T2DM, obesity, CAD, hypertension, and CKD were classified as the comorbidity group*.

* *p < 0.05*.

*** *p < 0.001*.

Table 4 presents the incidence, as well as the crude and aHR of hyperlipidemia among IBD patients with and without treatment compared to those without IBD. Compared to the control cohort, the IBD cohort without treatment had a 2.20-fold higher risk of hyperlipidemia (95% CI, 2.05–2.36). Stratification of each type of IBD treatment suggested that immunomodulators, aminosalicylates, and corticosteroids could significantly decrease the risk of hyperlipidemia (aHR, 0.29; 95% CI, 0.18–0.49, aHR, 0.43; 95% CI, 0.34–0.55, aHR, 0.45; 95% CI, 0.42–0.49, respectively) compared to that of patients with IBD without treatment. Further stratification according to surgical treatment for IBD showed that surgical treatment could reduce the risk of hyperlipidemia (aHR, 0.32; 95% CI, 0.20–0.51).

**Table 4:** Incidence, crude, and adjusted hazard ratio (aHR) of hyperlipidemia compared among inflammatory bowel disease (IBD) patients with and without IBD treatment compared to non-IBD controls.

**Variables**	***N***	**Event**	**PY**	**Rate^#^**	**Crude HR (95% CI)**	**Adjusted HR^*†*^ (95% CI)**	**Adjusted HR^*†*^ (95% CI)**
Non-IBD controls	14,524	2,345	107,920	21.7	1 (Reference)	1 (Reference)	
IBD without anti-IBD treatment	4,425	1,115	24,689	45.2	2.07 (1.92, 2.22)^***^	2.20 (2.05, 2.36)^***^	1 (Reference)
**IBD with anti-IBD treatment**							
Immunomodulators	138	15	1,171	12.8	0.59 (0.36, 0.98)^*^	0.65 (0.39, 1.08)	0.29 (0.18, 0.49)^***^
Aminosalicylates	478	68	2,937	23.2	1.06 (0.83, 1.35)	0.95 (0.74, 1.21)	0.43 (0.34, 0.55)^***^
Monoclonal antibody and small molecule	5	0	26	23.2	-	-	-
Corticosteroids	9,478	1,729	77,287	22.4	1.03 (0.97, 1.10)	0.99 (0.93, 1.05)	0.45 (0.42,0.49)^***^
IBD without surgical treatment	14,312	2,910	104,631	27.8	1.28 (1.21, 1.35)^***^	2.20 (2.05, 2.36)^***^	1 (Reference)
Surgical treatment	212	17	1,479	11.5	0.53 (0.33,0.85)^**^	0.69 (0.43, 1.12)	0.32 (0.20, 0.51)^***^

# *Incidence rate, per 1,000 person-years; Crude HR, crude hazard ratio*.

† *Multivariable analysis including age, sex, and comorbidities of T2DM, obesity, CAD, hypertension, and CKD*.

* *p < 0.05*.

** *p < 0.01*.

*** *p < 0.001*.

### Effects of IBD Medications on Lipogenesis-Related Gene Expression

We assessed the influence of corticosteroids (budesonide, prednisolone, methylprednisolone, and hydrocortisone), immunomodulators (azathioprine, methotrexate, cyclosporine, tacrolimus, and 6-mercaptopurine), and aminosalicylates (sulfasalazine, balsalazide, and 5-aminosalicylate) on hepatic gene expression. The concentrations were adjusted according to the maximum plasma or serum drug concentrations as they exhibit hepatotoxicity (35–47). We tested the toxicity of the drugs in a cell viability assay using human hepatoma HepaRG cells. The concentrations of corticosteroids were 0.01, 1.13, 0.72, and 1.42 μM for budesonide, prednisolone, methylprednisolone, and hydrocortisone, respectively; those of immunomodulators were 0.15, 1.77, 1.91, 0.03, and 0.77 μM for azathioprine, methotrexate, cyclosporine, tacrolimus, and 6-mercaptopurine, respectively; and those of aminosalicylates were 61.5, 1.40, and 60.1 μM for sulfasalazine, balsalazide, and 5-aminosalicylate, respectively.

**Figure 2:**
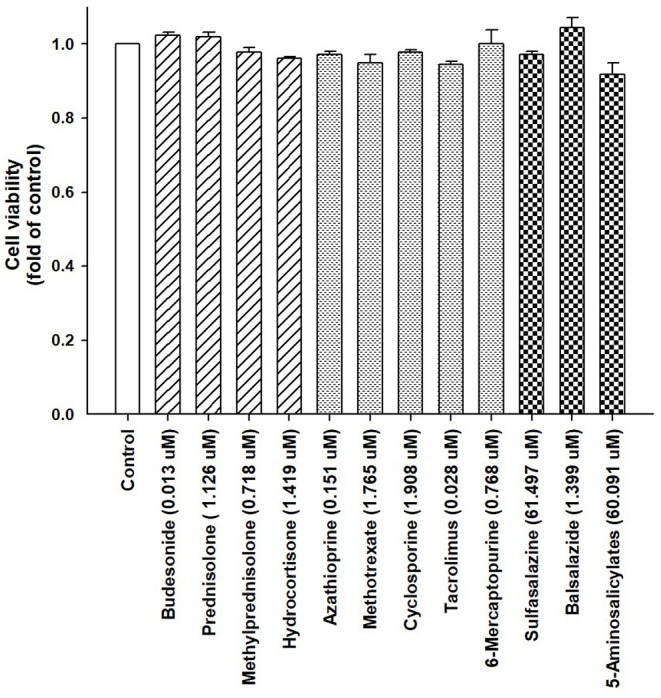
Viability of HepaRG cells following exposure to IBD medications. HepaRG cells were exposed to corticosteroids (budesonide, prednisolone, methylprednisolone, and hydrocortisone), immunomodulators (azathioprine, methotrexate, cyclosporine, tacrolimus, and 6-mercaptopurine), and aminosalicylates (sulfasalazine, balsalazide, and 5-aminosalicylate) for 48 h. Cell viability was monitored by cellular acid phosphatase activity using *p*-nitrophenylphosphate as a substrate. The data are shown as the mean ± SE (error bars) (*n* = 4).

**Figure 3:**
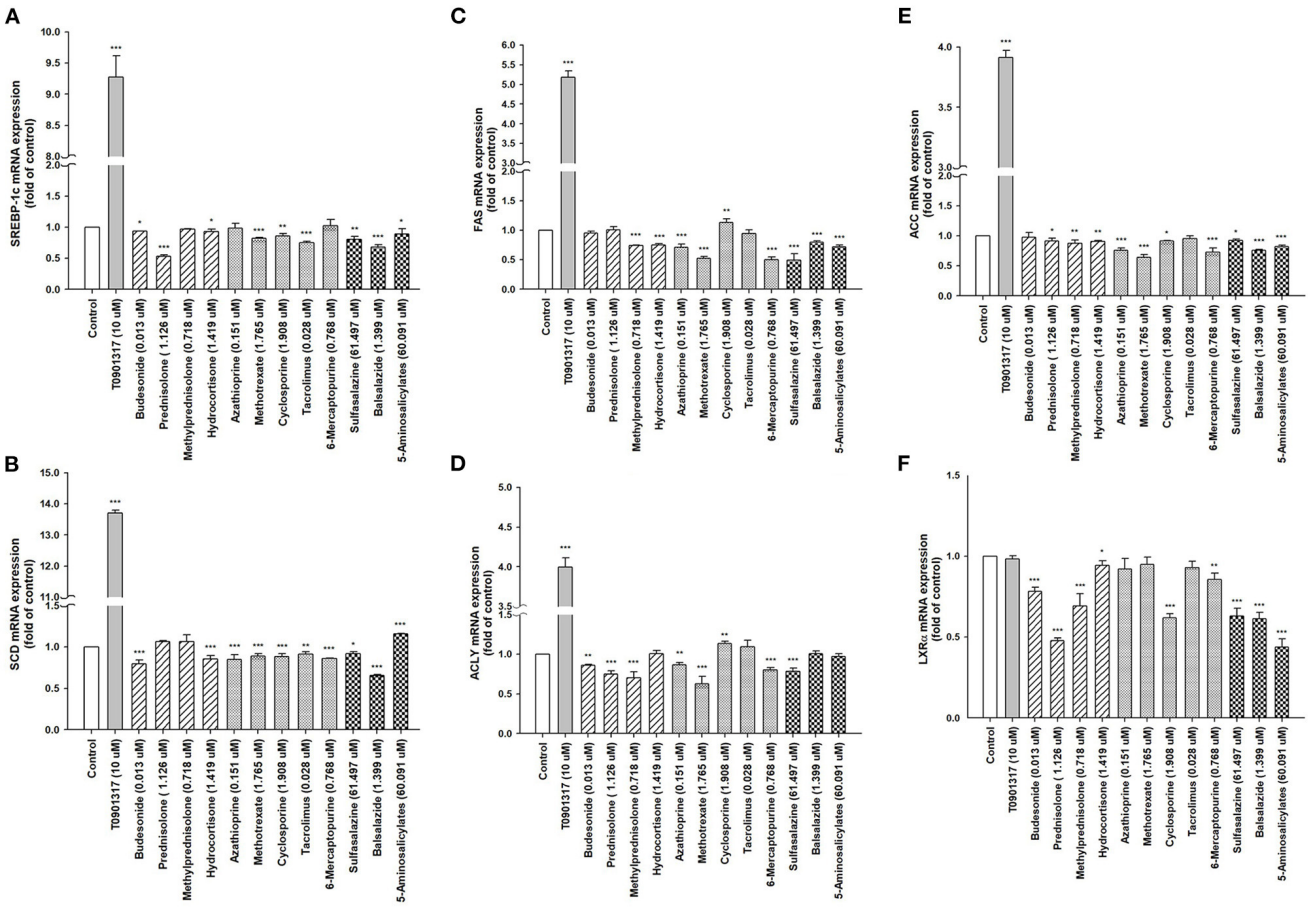
Expression of hepatic lipid metabolism-related genes following treatment with T0901317 and IBD medications. Differentiated HepaRG cells were treated for 48 h with T0901317 (10 μM), corticosteroids (budesonide, prednisolone, methylprednisolone, and hydrocortisone), immunomodulators (azathioprine, methotrexate, cyclosporine, tacrolimus, and 6-mercaptopurine), and aminosalicylates (sulfasalazine, balsalazide, and 5-aminosalicylate). Following treatment, mRNA was extracted and the expression levels of **(A)** *SREBP-1c*; **(B)** *SCD*; **(C)** *FAS*; **(D)** *ACLY;* **(E)** *ACC*; and **(F)** *LXR*α were analyzed by qRT-PCR. Values were normalized to the expression of β*-actin*, with the levels of DMSO-treated cells set at 1. Results are expressed as the mean ± SE (*n* = 3). *P < 0.05; **P < 0.01; ***P < 0.001 compared with cells treated with DMSO.

The cells were treated with IBD medications for 48 h, and the ACP assay was used to assess the cell viability. Our results indicated that the cell viability from each drug remained >95% compared with the control group (Figure 2). Moreover, the expression of lipogenesis-related hepatic genes, such as *SREBP-1c, SCD, FAS, ACLY, ACC*, and *LXR*α were assessed using RT-PCR in differentiated HepaRG cells treated with IBD medications for 48 h. The total RNA was extracted, and gene expression was analyzed. *SREBP-1c*, a transcription factor that regulates lipid homeostasis by modulating the expression of a series of target lipogenic genes (33), is mainly distributed in the liver and participates in hepatic fatty acid synthesis by upregulating the expression of downstream genes. T0901317, a synthetic *LXR*α agonist, significantly induced the expression of these target genes. However, the expression levels of *SREBP-1c, SCD, FAS, ACLY, ACC*, and *LXR*α were significantly lower in most treatment groups than the untreated groups (Figures 3A–F). IBD medications reduce the risk of hyperlipidemia and partially reduce the expression of hepatic genes involved in lipogenesis, resulting in improved blood lipid profiles.

## Discussion

To the best of our knowledge, this was the first study to report the correlation of IBD medications and risk of hyperlipidemia in patients with IBD, particularly in Asian populations. This study assessed 23 million people living in Taiwan using the NHIRD (47). The results show that the risk of hyperlipidemia was 2.18 times higher in patients with IBD than the general population after adjusting for confounding factors and comorbidities (*P* < 0.001). Moreover, this exclusive, nationwide population-based cohort study presented a comprehensive and complete assessment of an Asian population and discovered a significant association between IBD, IBD medications, and hyperlipidemia. We also found that without IBD therapies (medications and surgical treatment), the risk of hyperlipidemia was higher in patients with IBD than in the control cohort (aHR, 2.20; 95% CI, 2.05–2.36, *P* < 0.001). Furthermore, with IBD therapies (medications and surgical treatment), the risk of hyperlipidemia decreased in patients with IBD compared to those without IBD (all aHR ≤ 0.99, not significant). In addition, among patients with IBD, the risk of hyperlipidemia significantly decreased in those that received IBD treatment compared to those that did not (all aHR ≤ 0.45, *P* < 0.001). Finally, IBD medications in hepatocyte-derived HepaRG cells significantly reduced the expression of lipogenesis-related genes, such as *SREBP-1c, SCD, FAS, ACLY, ACC*, and *LXR*α, contributing to improved blood lipid profiles. These findings provide valuable insights for clinical physicians regarding the benefits of IBD therapies for improving blood lipid profiles and CVD prognosis, as well as reducing the risk of CVD through blood lipid control. Our study clarifies the impact of IBD medications on the development of hyperlipidemia, and the decreased risk of hyperlipidemia in patients receiving treatment may be due to drug-induced downregulation of lipogenic genes, especially the master lipogenic transcription factors, *SREBP-1c* and *LXR*α.

Our analysis of 29,048 people (14,524 with IBD and 14,524 without) with a mean follow-up period >7 years showed an overall significant increase in risk of hyperlipidemia compared with the general population (aHR, 2.18). Moreover, males, patients >65 years of age, and those with comorbidities were at higher risk than those in the general population matched by age, sex, and comorbidities of T2DM, obesity, CAD, hypertension, and CKD. As such, these groups may require early and aggressive intervention for CVD risk management. Furthermore, a significant association was observed between hyperlipidemia and IBD; however, patients with IBD that received IBD therapy had a decreased risk of hyperlipidemia compared to those that did not receive therapy.

Several large meta-analyses and cohort studies have reported increased rates of CVD in patients with IBD. However, hyperlipidemia was less likely to be assessed among such patients, which could result in the silent progression of atherosclerosis and vascular defects. Moreover, acute myocardial infarction (AMI) was found to be higher in patients with IBD than in those without (OR, 12.05; 95% CI, 11.16–13.01), as was the rate of VTE (12, 18). A prospective study of 24 patients with IBD who had undergone surgical treatment, exhibited increased levels of HDL-C, TC, and LDL-C in patients in remission compared to those with recurrent active IBD (48). In another retrospective study, TC and LDL-C levels were significantly lower in patients with IBD after restorative proctocolectomy than in control subjects (49). These findings may be due to malabsorption from accelerated transit and the exclusion of the terminal ileum caused by the covering ileostomy (49). TC and HDL-C levels were significantly lower, TG levels were significantly higher in patients with IBD compared to controls were also reported (50). Ripollés Piquer et al. suggested that the levels they observed corresponded with those of atherogenesis and may contribute to the development of CVD (24). Nevertheless, more large-scale and epidemiological studies are warranted to evaluate lipid alterations in patients with IBD more in depth.

Lipid profile alterations in patients with IBD have been presented in previous studies. First, Becker and colleagues (51) showed an association of decreased TC levels and increased TG levels with intestinal resection in patients with CD in a study of 22 children with CD and 10 healthy controls. Moreover, a US study (22) concluded that patients with IBD have lower TC and HDL-C levels and higher LDL-C and TG levels compared to controls from the NHANES 2005–2006 database. Another report showed that patients with IBD have lower TC and LDL-C levels compared to control subjects due to systemic inflammation from reduced lipoprotein and hepatic lipase activity and decreased cholesterol absorption (52). The conflicting results of these studies may be partially explained by the differences in study population, methodology, data interpretation, duration of sampling, and baseline characteristics of the study samples (21, 22, 50). While our findings are not consistent with all studies, we performed an in-depth analysis of different treatment modalities over a prolonger follow-up period. Moreover, our study was based on a nationwide population database rather than a single institution.

No study has previously reported the correlation of IBD therapies with hyperlipidemia. We found that IBD treatments can prevent patients from developing hyperlipidemia. Thus, while IBD can neither be prevented nor cured, hyperlipidemia can be partially prevented by appropriate interventions such as diet control, regular exercise, and reduced body weight. In view of hyperlipidemia-related CVD, physicians who participate in the long-term care of patients with IBD should consider aggressive modification of risk factors associated with hyperlipidemia. Finally, we conclude that the association of IBD and hyperlipidemia remains to be explored due to conflicting results of each study.

The association between IBD and the increased risk of CVD has been previously reported in a systemic review. The review observed 6,478 coronary events in 123,907 patients with IBD and found a 19% higher risk of CVD in those with IBD (53). Additionally, patients with IBD exhibited a higher risk of carotid atherosclerotic plaque, warranting evaluation of carotid intima-media thickness through ultrasonography for this high-risk subpopulation. Moreover, Kristensen et al. (13) found that compared with controls, patients with IBD presented an increased risk of stroke [relative risk (RR), 1.15; 95% CI, 1.05–1.27] and hospitalization due to heart failure (RR, 1.37; 95% CI, 1.26–1.49) (13). Since, hyperlipidemia is a major risk factor for stroke, which is a leading cause of death (54, 55), may play a role in this situation. In addition, a case-control study reported an increased risk of stroke in patients with IBD (aOR, 2.93; 95% CI, 1.44–5.98) especially in those younger than 50 years of age (56). Thus, the periodic and continuous monitoring of CVD risk in this population is necessary. However, no specific guidelines exist for patients with IBD regarding the prevention and management of CVD (9, 53). Although the current American Heart Association/American College of Cardiology guidelines on blood cholesterol management showed that chronic inflammatory disorders increase the risk of CVD, patients with IBD have not been specifically assessed (57).

Our findings show that patients with IBD were likely to have more comorbidities. For example, several pathophysiological factors are involved in the development of hyperlipidemia in these patients and may increase the risk of CVD. However, lipid profile monitoring in IBD patients is not routinely performed in Taiwan. Additionally, awareness of hyperlipidemia is lower than that of diabetes and hypertension; therefore, hyperlipidemia is diagnosed only when physical complications, such as CVD accompanied by chest pain (58). Thus, hyperlipidemia is rarely managed in patients with IBD. We suggest that clinicians perform blood lipid profiling for patients with IBD due to their increased risk of hyperlipidemia to provide suitable treatment and complete medical care.

Although a previous report showed that body mass index and blood lipid levels are often low in patients with IBD due to poor absorption of bile during active inflammation and after surgery (59). Our data showed that obesity is pronounced in these patients, which may further increase the risk of hyperlipidemia. Another report revealed that the inflammation-based increase of TGs is caused by increased hepatic lipoprotein production and decreased lipoprotein clearance. TGs are mediated by apolipoprotein C-III, which is a main risk factor for atherosclerogenesis (60). Several studies have indicated that chronic inflammation is associated with altered blood lipid profiles and CVD and generates inflammatory cytokines such as *TNF*α, reduces *NOS* expression, decreases NO bioavailability, impairs endothelium vasodilation, and increases cell adhesion molecules and stimulating leukocyte migration, therefore promoting atherosclerosis (61). Inflammatory cytokines, such as *TNF, IL-6*, and *IFN-*γ may downregulate lipolytic enzyme activity (8); however, the pathophysiological activities in IBD are more complex because of the chronic inflammation, malnutrition, and lipid malabsorption due to intestinal damage or resection (25).

Previous study found that low TC and LDL-C levels were correlated with systemic inflammation and CRP levels in IBD (52). Meanwhile, one study that observed a significant association of low TC and high TGs levels with disease severity failed to present any association with increased levels of inflammatory biomarkers (CRP, ESR, and albumin) (21). They reported increased risk of CVD in patients with IBD along with lower TC levels and chronic inflammation reflects a complicated and poorly understood pathogenic mechanism, described as a “lipid paradox” or dyslipidemia. A better understanding of dyslipidemia in IBD will help prevent and manage atherosclerosis and CVD.

We found that, each group of patients that received IBD medications showed a significant decrease in risk of hyperlipidemia (all aHR < 0.46) after adjusting for age, sex, and comorbidities with T2DM, obesity, CAD, hypertension, and CKD. Whether IBD medications play an important role in reducing the risk of hyperlipidemia and CVD had not been previously elucidated. Moreover, although it has been reported that corticosteroids may increase the risk of atherosclerosis and acute coronary syndrome resulting from hypertension, hyperlipidemia, and thromboembolism (11, 26), they overall reduce the risk of CVD through inhibition of inflammatory response. In the current study, we also found that corticosteroids may inhibit hepatic lipogenic gene expression. Thus, the contribution of corticosteroids to the reduced risk of CVD remains controversial. Nevertheless, methotrexate has been reported to rarely induce steatohepatitis associated with acute coronary syndrome and thromboembolic events (62, 63). We found that among the immunomodulators, methotrexate and 6-mercaptopurine inhibited the expression of lipogenic genes. Moreover, cyclosporine was associated with increased risk of acute coronary syndrome and heart failure (64). In addition, 5-aminosalicylates, a standard first-line treatment for IBD, may be used to prevent CVD and VTE in patients with IBD (14, 65, 66) due to their anti-inflammatory effects and ability to inactivate platelets. Moreover, we found that most 5-aminosalicylates were shown to inhibit lipogenic gene expression.

Increased carotid intimal thickness, wall stiffness, and endothelial dysfunction observed in patients with IBD may be due to the increase in circulating inflammatory cytokines and CRP (67). IBD medications are beneficial for their dual effects of decreasing the expression of lipogenic genes and treating IBD symptoms; however, the net effects of IBD medications on the lipid profile in terms of CVD risk remain uncertain. Thus, the metabolic profiles of patients with IBD should be considered when making therapeutic decisions. Nevertheless, prospective studies are needed to evaluate the dual effects of IBD medications in the general IBD population.

We performed a longitudinal population-based cohort study with cases and controls matched for sex and age to explore the possible correlation of hyperlipidemia and IBD in Taiwan. We also evaluated the effect of IBD medications on the risk of developing hyperlipidemia. Nevertheless, our study had several limitations. First, the database we used may have misclassified IBD and hyperlipidemia, limiting the reliability and validity. However, in Taiwan, universal health insurance is distributed, and as a result, a peer review system is enacted by specialists to reduce the possibilities of false positives. Second, several potential risk factors of hyperlipidemia, such as smoking habits, alcohol intake, body mass index, lifestyle and dietary habits, and family history of hyperlipidemia were not included in this study as they are not provided in the NHIRD. In addition, the risk of hyperlipidemia according to the severity of IBD could not be estimated, as the database had no information regarding the severity of IBD, such as Harvey-Bradshaw Index and Mayo score. Third, this claim database was initially created for charging purposes, and as some information was anonymized, we could not contact the patients directly to get individual data from them. Fourth, several clinical laboratory data were not included in this database, and as such, we could not assess the degree of hyperlipidemia in patients with IBD. For example, serum TC, TGs, HDL-C, and LDL-C. Cases of hyperlipidemia were defined indirectly according to the physicians' records based on lipid levels under IBD medication therapy. In this study, both IBD and hyperlipidemia were accurately analyzed and coded (ICD-9-CM codes) by specialists according to the standard symptomatic criteria by considering the normal side effects, signs, research facility information, and imaging findings. Additionally, this study reduced the confounding effect of medications by adjusting for comorbidities. However, more information should be obtained from other databases to conduct a comprehensive prospective study or randomized controlled trial to further investigate the relationships of IBD, IBD medications, and hyperlipidemia. Most crucially, evidence derived from a retrospective cohort study is typically lower in statistical quality because of numerous sources of inherent bias, including the classification bias. However, the NHI program has a high coverage rate, and medical reimbursement specialists and peer reviewers scrutinized all insurance claims, ensuring that the diagnoses and coding of diseases were highly reliable in the NHIRD. The classification bias was also supposed to be non-differential, and this should not invalidate our result. Primary human hepatocytes (PHHs) are a well defined *in vitro* hepatic model to predict drug responses with respect to metabolism and toxicity, based on the proper maintenance of metabolism, transport, and biological signaling pathways. However, the significant variability among individuals and high cost of PHHs has led to the development of alternative cell line models. Thus, HepaRG cells have emerged as a promising alternative to PHHs as an *in vitro* model, as this cell model, upon reaching phenotypic maturity, can grow to confluence and differentiate over 4 weeks (from progenitor cells) into cocultures of hepatocyte-like and cholangiocyte-like cells (68). However, it must be kept in mind that cell lines do not behave identically to primary cells and do not necessarily completely replace and reflect the effects and outcomes of primary cells.

This study had several strengths. First, we used a nationwide, population-based cohort of patients with anonymized data to minimize selection bias. Additionally, we evaluated the effect of IBD and IBD medications on the risk of hyperlipidemia over a prolonged follow-up period. Moreover, by adjusting for age and sex in a 1:1 ratio, we accounted for confounders that may affect the occurrence of hyperlipidemia. Finally, although detection bias may have occurred if patients had more hospital visits than the control population by increasing the possibility of detecting hyperlipidemia, the risk of hyperlipidemia was still increased 2.2-fold in patients with ≥2 hospital visits per year. Overall, this is the first study to investigate the IBD-associated risk for developing hyperlipidemia and evaluate the effects of IBD medications on lipogenic gene expression.

## Conclusion

In conclusion, patients with IBD have a significantly increased risk of developing hyperlipidemia compared to non-IBD controls. However, under all IBD medications and surgical treatment, patients with IBD experienced a reduced risk of hyperlipidemia compared with non-treated IBD patients. We further confirmed that the effects of IBD medications on the decreased expression of lipogenic-related genes contributed to the beneficial effects on the blood lipid profiles. Therefore, regular monitoring of blood lipid levels should be considered for the early detection of hyperlipidemia in patients with IBD, especially in elderly patients, to decrease the risk of CVD. Large, population-based studies or randomized clinical trials are warranted to confirm our observation that IBD and IBD medications play an important role in the development of hyperlipidemia. In addition, early detection, monitoring, aggressive, and comprehensive treatment for metabolic disturbances, and alleviation of acquired risk factors are highly recommended. Further investigations into the possible biological and pathological mechanisms underlying the relationship between hyperlipidemia and the use of IBD medications are essential.

## Data Availability Statement

The raw data supporting the conclusions of this article will be made available by the authors, without undue reservation.

## Ethics Statement

The NHIRD encrypts patient personal information to protect privacy and provides researchers with anonymous identification numbers associated with relevant claims information, including sex, date of birth, medical services received, and prescriptions. Therefore, patient consent is not required to access the NHIRD. This study was approved to fulfill the condition for exemption by the Institutional Review Board (IRB) of China Medical University (CMUH-104-REC2-115-R5). The IRB also specifically waived the consent requirement.

## Author Contributions

NT, T-YW, Y-JF, and Y-PL: conceptualization, methodology, investigation, supervision, and funding acquisition. C-LL, C-JW, and C-YH: software and formal analysis. NT and Y-PL: validation. C-LL, C-YH, and Y-PL: resources and data curation. C-YH and Y-PL: project administration. All authors: writing—original draft preparation, writing—review and editing, and visualization. All authors contributed to the article and approved the submitted version.

## Funding

This study was supported by the Ministry of Science and Technology, Taiwan, R.O.C. (MOST110-2320-B-039-016-MY3), China Medical University, Taichung, Taiwan (CMU110-MF-29), China Medical University Hospital, Taichung, Taiwan (DMR-111-105; DMR-111-228), partially supported by the Taiwan Ministry of Health and Welfare Clinical Trial Center (MOHW110-TDU-B-212-124004), Taichung Tzu Chi Hospital, Buddhist Tzu Chi Medical Foundation (TTCRD111-31), Show Chwan Memorial Hospital, Changhua, Taiwan (SRD-110027).

## Conflict of Interest

The authors declare that the research was conducted in the absence of any commercial or financial relationships that could be construed as a potential conflict of interest.

## Publisher's Note

All claims expressed in this article are solely those of the authors and do not necessarily represent those of their affiliated organizations, or those of the publisher, the editors and the reviewers. Any product that may be evaluated in this article, or claim that may be made by its manufacturer, is not guaranteed or endorsed by the publisher.

## Abbreviations

ACLY: ATP citrate lyase
ACP: acid phosphatase
AMI: acute myocardial infarction
aOR: adjusted odds ratio
CAD: coronary artery disease
CD: Crohn's disease
CI: confidence interval
CKD: chronic kidney disease
COPD: chronic obstructive pulmonary disease
CRP: C-reactive protein
CVD: cardiovascular disease
DMSO: dimethyl sulfoxide
ESR: erythrocyte sedimentation rate
FAS: fatty acid synthase
HDL-C: high density lipoprotein cholesterol
HR: hazard ratio
IBDs: inflammatory bowel diseases
ICD-9-CM: International Classification of Diseases, Clinical Modification
IFN-γ: interferon γ
IL-6: interleukin 6
LDL-C: low density lipoprotein cholesterol
LHID: Longitudinal Health Insurance Database
LXRs: Liver X receptors
NAFLD: non-alcoholic fatty liver disease
NHIRD: National Health Insurance Research Database
NHRI: National Health Research Institutes
NO: nitric oxide
NOS: nitric oxide synthase
PNPP: para-nitrophenylphosphate
qRT-PCR: quantitative real-time polymerase chain reaction
RR: relative risk
SCD: stearoyl CoA desaturase
SD: standard deviation
SE: standard error
SREBP-1c: sterol regulatory element binding protein 1c
T2DM: type 2 diabetes mellitus
TC: total cholesterol
TGs: triglycerides
TNF: tumor necrosis factor
UC: ulcerative colitis
VTE: venous thromboembolism.
